# Collaborative learning in small groups in an online course – a case study

**DOI:** 10.1186/s12909-022-03232-x

**Published:** 2022-03-10

**Authors:** Mildrid Jorunn Haugland, Ivar Rosenberg, Katrine Aasekjær

**Affiliations:** 1 Faculty of Health and Social Sciences, Department of Health and Functioning, Western Norway University of Applied Sciences/Høgskulen på Vestlandet, Inndalsveien 28, 5063 Bergen, Norway; 2 Faculty of Health and Social Sciences, Academic Affairs, Western Norway University of Applied Sciences/Høgskulen på Vestlandet, Inndalsveien 28, 5063 Bergen, Norway; 3 Faculty of Health and Social Sciences, Department of Health and Caring Sciences, Western Norway University of Applied Sciences/Høgskulen på Vestlandet, Inndalsveien 28, 5063 Bergen, Norway

**Keywords:** Collaborative learning, Small group, Online course, Case study, Qualitative study, Working process

## Abstract

**Background:**

The ability to learn collaboratively and work in teams is an essential competency in both educational and healthcare settings, and collaborative student activities are acknowledged as being an important part of the pedagogical approach in higher education and teaching. The course that was the focus of this research, a 15-ECTS-credit online course in philosophy of science, ethics, and research methods, was offered online as part of 11 master’s-level health programmes at a university in Norway. Collaborative learning in combination with digital teaching tools was the preferred pedagogical approach in the online course. The aim of the study was to describe, explore and discuss how the students collaborated in small groups in an online course to learn.

**Methods:**

We performed six focus groups and 13 individual interviews from February 2018 to May 2019, conducting a qualitative case study with a content analysis of the data collected. The participants were master students in the same faculty at a university in Norway. All the included participants had fulfilled the 15 ECTS credit course.

**Results:**

Our study revealed that the collaboration in small groups resulted in three different working processes, depending on the students’ ability to be flexible and take responsibility for their own and common learning. The three different working processes that emerged from our data were 1. joint responsibility – flexible organization; 2. individual responsibility – flexible organization; and 3. individual responsibility – unorganized. None of the groups changed their working process during their course, even though some experienced their strategy as inadequate.

**Conclusions:**

Our study showed that despite similar factors such as context, assignments and student autonomy, the students chose different collaboration strategies to accomplish the online course learning objectives. Each group chose their own working process, but only the strategy 1. joint responsibility – flexible organization seemed to promote collaboration, discussion, and team work to complete the complex assignments in the online course. The result from our study may be helpful in designing and planning future online courses; hence online learning requires a focus on how students collaborate and learn online, to gain knowledge and understanding through group discussion.

## Background

Education is increasingly being offered online, and there is growing demand in higher education for online studies and courses using online resources in teaching and learning [1, 2]. E-learning worldwide is expected to account for 30% of all educational provision [3, 4]. This has led to an increase in educational provision offered online (all or in part) and the need for improved articulation between technology and pedagogy in higher education [5]. However, even though online teaching is in demand in both educational institutions and among students themselves, studies show that the ability to complete online education is reduced, compared to face-to-face teaching [6–8]. One suggestion for reducing dropout rates is to have a mix of online and face-to-face courses in study programmes [9].

Collaborative learning (CL) and teamwork skills developed through working in groups are important competencies for healthcare workers [10]. Group work is therefore a pedagogical method that is widely used in health and social science education (e.g., problem-based learning (PBL), team-based learning (TBL) and simulation training), and much research has been carried out into these pedagogical methods [11]. Collaborative learning is defined as teaching or learning activities that promote an individual’s own learning and that of others in small groups (two to five students) or collaboration (cooperation) to achieve common goals [12, 13]. In CL, learning is a dynamic process involving interaction between individual students’ drive to learn and a social activity in a specific context [14–16]. While students in CL are mutually dependent on one another, to be able to discuss and reflect and thus achieve a deeper understanding of the subject matter, they learn from one another through reflection in the situation and on the situation [17–20].

Research has shown which design and group working process factors that can positively influence collaboration within groups. Design factors include group size (three–five), group composition and the nature of the assignments [20]. Positive interdependence and individual accountability are important factors for group working processes [20, 21]. In Norway, two reports [22, 23] have concluded that students’ learning depends on how digital tools are implemented and how these tools are used within the pedagogical situation. It is therefore important to look at which factors are important for CL to achieve discussion and reflection, thereby facilitating in-depth learning and collaboration, when students complete assignments. An understanding of the factors that facilitate students’ collaboration is critical to understanding how this approach to learning can be used more effectively in online courses in higher education.

The aim of the study was to describe, explore, and discuss how students undertaking an online course collaborated in small groups. Our research question was therefore: How did the students collaborate in small groups to achieve learning online?

We adopted a social-constructivist approach to learning in this study. This approach emphasizes that understanding CL and the various roles students have in the learning process requires examining the interaction that is taking place and the context of this interaction [16, 24–27]. In this approach learning is seen as a dynamic social process where increased knowledge is considered a consequence of social interaction.

When the study was conducted, all master’s students at the Western Norway University of Applied Sciences, Faculty of Health and Social Sciences, had completed an online course in the philosophy of science, ethics, and research methods (MaMet).

Each programme of study created in MaMet its own assignments, making them relevant to their specific professions/programmes and ensuring an appropriate level of difficulty for all students. The courses were run at different time periods for each programme and by teacher associated with that specific programme. In some programmes, this was the only online course, others programmes had several online courses. A key learning method in this course was CL in small groups, through written assignments and peer reviewing of fellow students’ assignments.

### Description of MaMet

The online course in philosophy of science, ethics, and research methods was completed in 11 master’s programmes, and each study programme was responsible for administering the course by facilitating, guiding and following the students over the course of this module in their master’s programme. The number of students enrolled in each programme differed, ranging from 12 to 90. MaMet is grounded on a small-scale online course, where there is planned discussion and feedback among teachers and students throughout the whole course. Student activity and CL are a cornerstone in the pedagogical and didactical thinking in MaMet, and work in small groups, with assignments, is the most prominent methodology in implementation of the course. By completing every problem-based assignment in the course, students gain the knowledge and skills to be able to design their own study protocol. The digital resources in MaMet include design focusing on learning outcomes, enabling students to develop the ability to understand and perform research projects.

Each small group in the study consisted of the same students throughout the whole course and included three to five students. Group members were responsible for the work processes of the group, how task problems were solved, when and how the group members met and the collaborative structure within the group. Group composition and the content of assignments were defined by the master’s programme. The groups did not have supervisors for small-group collaboration.

## Methods

This case study involved individual and focus group interviews with master’s students in the Faculty of Health and Social Sciences, to gain feedback about their experience of collaborative online learning in small groups in an online course. By conducting a qualitative case study, we were able to generate an in-depth understanding of a complex issue in a real-life context [28, 29].

The study was approved by the Norwegian Centre for Research Data (NSD 60336) and the academic institution. All the participants gave written consent after receiving written and oral information about the study and were given the option to withdraw from the study if they wanted to. All data were anonymized and kept confidential, in compliance with the ethical guidelines of the Declaration of Helsinki.

We conducted both focus-group and individual interviews because some participants felt that taking part in focus groups with their fellow students would be difficult as their experiences were connected to collaboration with peers. Differentiation of focus-group or individual interviews was done in collaboration with the participants, and with a focus on including students from all the master’s programmes who had completed the online course in philosophy of science, ethics, and research methods. Given that the participants completed the 15-ECTS programme at different points during the year, it was not possible to combine students from different programmes in the focus group interview.

### Participants and settings

Participants for the individual and focus group interviews were selected by means of purposeful sampling. This sampling method enabled us to include participants who could contribute information relevant to the aim of the study [28, 29]. Participants were master’s students who had participated in the 15-ECTS-credit online course in philosophy of science, ethics, and research methods. A total of 260 students completed the course, split into 65 small groups, and these students received a written invitation (via the online course) to participate in the study and an oral invitation (when attending lectures on campus). Thirty students from all 11 master’s programmes expressed an interest in taking part, and all 30 were included. We conducted six focus group meetings and 13 individual interviews. The 30 participants represented 25 different groups. Two of 30 participants were males, which reflects the overall gender distribution in the programmes. Each focus group consisted of between two and six students.

### Data collection

The same person (MJH) moderated all individual and focus group interviews, and IR co-moderated the focus group interviews conducted between February 2018 and May 2019. MJH had not been involved in development of the online course and had never met the students before. The focus group interviews were conducted face to face. Some individual interviews were conducted over the phone if that best suited the students. We conducted focus group interviews since our understanding is that students will be influenced by and have an influence on others present, providing a collection of rich and meaningful data [30, 31]. We believe that interaction through focus groups can inspire students to reflect and talk about the challenges associated with the topic. To facilitate this interaction, we conducted the focus group interviews in settings free of disturbance, on campus. The interviewer made all the arrangements regarding time and place, in agreement with the students.

The data collection was conducted within 2 months of the course ending. In this way, the students would still be able to remember their experiences while, at the same time, having a certain amount of distance from them. The themes in the semi-structured interview guide were as follows: a typical day (what activities/events took place and when); use of resources; motivation for online learning; collaboration in students’ respective small groups; and collaboration with teachers and fellow students. If any theme was not mentioned by the students during the conversation, the interviewer asked about it. The interviews lasted from 30 to 75 min, until data saturation occurred [31, 32]. The interviews were recorded and transcribed verbatim by MJH and KAA and approved by all the authors, ensuring that no essential information was lost during the transcription process.

### Data analysis

We used content analysis to analyse the data [30, 33], starting with all three authors reading and re-reading the interviews to get an overall impression of the data. Two of the researchers (MJH and KAA) worked separately and divided the text into units of meaning. They then grouped and coded these. This was done for each interview. We compared and discussed the codes across the interviews, before identifying categories. All interviews were then analysed again, with a focus on codes extracted to form categories. At the end of the analysis process, we created a condensed narrative with quotes, to illustrate what appeared in the categories. Relevant subthemes were identified to highlight key similarities and differences in the three main themes, based on what we found in the data. All three researchers discussed the sub-categories and further abstracted and reorganized these into themes and subthemes. Table 1 gives examples of the abstraction process from meaning units, code, and themes to subthemes. The condensed narrative formed the basis for the results presented.

**Table 1:** Example of the abstraction process; meaning units, code, themes, and subthemes

Meaning units	Code	Themes	Subthemes
“By collaborating online, we had to have strict rules within the group, so we didn’t spend time interrupting each other. We met on a regular basis since none of the group members lived in the same city, and it was great starting at times that matched our schedule. It made the work flexible. We solved all the assignments in collaboration, and all the members had a common responsibility for the assignments.” Interview 8 “We tried to distribute the task so that those who felt that they had not understood an area so well had to take on responsibility for that part of the task. We agreed that we had to do this for the sake of learning. Then we met again when we had worked on the task and discussed [it] and tried to put everything together. If there was anyone who disagreed with something, we changed it together.” Interview 16 “I experienced that I learned more by participating in discussions with my peers and solving tasks together than spending time alone with my books. You have to participate and take responsibility for your own learning to be able to contribute.” Interview 13 “We think it has been very educational, to be able to discuss with our fellow students the different tasks. That we collaborated on it, that we could discuss if things were unclear. Someone else in the group often had a different way of explaining it that made them finally understand it.” Interview 16	• Common understanding • Respective contribution • Regular meetings – flexible work • Collaboration and common responsibility • Loyal to the group • Learning in discussion and solving tasks • Took responsibility for their own and fellow students’ learning	1. Joint responsibility – flexible organization	1. Common understanding of the tasks 2. Common expectations clarified 3. Common and shared responsibility 4. Everyone was prepared 5. Flexible, open working processes 6. High degree of group loyalty and understanding 7. Shared responsibility for fellow students’ learning

## Results

**Fig. 1:**
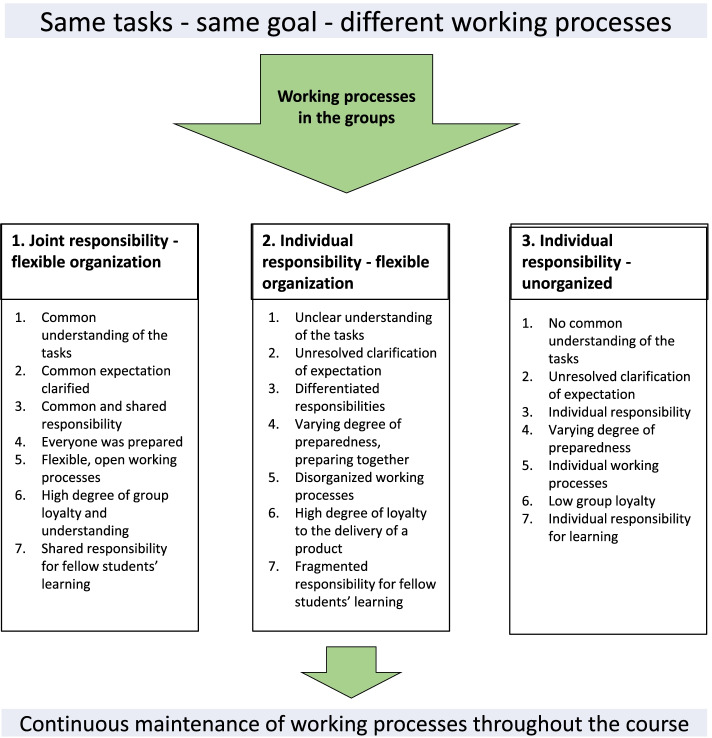
An overview of how the various groups organized their work

Our analytical process revealed themes and subthemes underpinning experiences essential to understanding how students collaborated in small groups in an online course. We found that when students were collaborating online in a group, the groups developed different strategies to solve the course assignments. All groups had the same goal for their work but used different working processes to reach that goal. Figure 1 summarizes the three different working processes that emerged: 1. joint responsibility – flexible organization; 2. individual responsibility – flexible organization; and 3. individual responsibility – unorganized.

The different working processes reflected the main characteristics of the group. We found seven subthemes that characterized the work process: understanding of the tasks, expectation of the group members, responsibility for the group work, preparedness for the group meetings, organization of the group work, group loyalty, and responsibility for fellow students’ learning. Each group seemed to maintain its working process throughout the online course, even if students told us that they experienced, as the work progressed, that there could be other and more appropriate ways of collaborating to complete the various assignments. The students’ explanation for not attempting to change their working process, was that they wanted to avoid conflicts and damage the atmosphere in the group. *There is no point in complaining, it will not solve the problem, only cause unpleasant feelings.” Interview 13.*

Regardless of which working processes the students engaged in, the students reported that group assignments were important for learning philosophy and methods relevant to science, and that problem-based assignments enabled them to use all the learning resources in the online course as the assignments were so closely linked to the learning resources. Furthermore, the students stated that it was important to continue the collaboration with the same students throughout the whole course. The topics were complex and difficult to understand, and by having the same group members, the working process was more predictable. The students also thought that the group sizes were appropriate.

### Joint responsibility – flexible organization

These students reported that they were prepared and informed about the assignments ahead of them. The work was characterized by joint responsibility and had a clear structure and framework to promote collaboration. The structure was such that there was still some flexibility, and it could be adjusted during the working process to suit the needs of the groups. There was a loyalty in relation to the work process to be carried out, and group members’ level of participation was high. This working process was characterized by discussion and reflection on the assignments and an understanding that learning was promoted through the group working process. The groups worked independently, with less need for input from the teachers.

*“By collaborating online, we had to have strict rules within the group, so we didn’t spend time interrupting each other. We met on a regular basis since none of the group members lived in the same city, and it was great starting at times that matched our schedule. It made the work flexible. We solved all the assignments in collaboration, and all the members had a common responsibility for the assignments.” Interview 8.*

We found that with this model (joint responsibility – flexible organization), the groups collaborated in different ways to solve the assigned problem. In some of the groups, the students started the work together, distributing the work among group members, and then came together to discuss progress, distributing further work within the group as required. The work was characterized by short meetings to clarify a common understanding and to distribute responsibilities. Other groups spent a long time on the working processes and did all the work together online, focusing on common understanding.

*“We met every day at 10. Sometimes we discussed in a videoconference, sometimes just chatting, or emailing. We divided the task and had individual responsibility. But at the same tame we gave both written and orally feedback on fellow students work. So, we had an individual part, but were involved in the whole task” Interview 13*

Despite some differences in working processes, the main characteristic of collaboration was that the groups had a mutual aim (i.e., that all students in the group had a common understanding of the task in hand and respective contributions), and they consequently took responsibility for their own and fellow students’ learning.

### Individual responsibility – flexible organization

In this working process, the rationale for the online course and how the assignments would contribute to the students becoming qualified professionals was less clear to the students. Consequently, the group members had varying degrees of preparedness, and use of the digital learning resources was more fragmented and limited to the assignments that the group had to complete.

*“I don’t understand why I need philosophy and method in my profession. I am not going to be a scientist, I am supposed to be [an anaesthetic nurse, operating room nurse, intensive care nurse]. I really don’t see the point. I believe only those who actually want to do a master thesis should do this.” Interview 7.*

When these students met for the first time, they were not prepared. They organized the work by dividing the assignments into smaller sections, and they completed different sections of the overall assignment separately, bringing their respective contributions together to assemble an answer at the end. In this collaboration process, work was also distributed within groups differently. Assignments were tackled either by distributing an entire task to each of the members or by dividing the relevant task into smaller units so that each student contributed to each task. In both approaches, individuals had responsibility for completing part of the overall task.

*“We organized the group by delegating one assignment to two group members at a time. Meaning that two students had the main responsibility for one assignment, and the other members gave some comments on the work. This gave us a greater flexibility and not so much work. But I don’t have so much knowledge and control over the themes that I didn’t have responsibility over.” Interview 10.*

In this working model (individual responsibility – flexible organization), there was less focus on meeting one another to discuss the work in progress, but rather, a greater focus on delivering a product – the assignment. Discussion about the product was characterized by whether the assignment contained what it needed for it to be approved, and there was less discussion of the group members’ understanding of the task. Group members’ input tended to be presented to the group individually, rather than during group discussion, which could result in a situation with two conflicting inputs. It was thus up to the person responsible for delivering the assignment to assess which input should be considered or whether the input should be considered at all.

*“I wanted the answer to be the best possible. Everyone did their part and pasted it into the document. And then almost nothing happened. We were left with a fragmented answer with many yellow boxes and comments on the page. I had to take responsibility for the last bit to put [it all] together and make it a whole. Students stopped contributing when they felt they had finished with their part Often I had the impression that they did not care and that the focus was elsewhere.” Interview 12*

### Individual responsibility – unorganized

This working process had no group structure because how the work was organized depended on individuals taking responsibility on behalf of the group. The main characteristic of this working process was that the student(s) who took responsibility were the same throughout the online course, and these students were highly motivated. They recognized the importance of learning and therefore knew how to go about solving the assignment. This working process lacked structure and organization, and there was an absence of cooperation and discussion. Most of the group members gave their input only when the product was available.

*“It became an extra workload on my account, because I felt that I had to do the assignments so that we could deliver a product. You depend on those who are supposed to participate to take responsibility for their own learning. There is no point in complaining, it will not solve the problem, only cause unpleasant feelings.” Interview 13.*

In this working process, one or two students in each group were responsible for the work. Group members who did not take any responsibility only attended group meetings when these did not conflict with their own needs or priorities. Group dynamics existed to a limited extent because the groups were characterized by an absence of participation, discussion and flexibility among group members. Those individuals who took responsibility had little opportunity to assume responsibility for anything other than their own learning and understanding, but they expressed the desire for a community where they could share knowledge through discussion and reflection and thus enable everyone to gain greater knowledge.

*“It gave an unpleasant feeling – the responsibility of doing the assignments on your own. I didn’t feel we were a group who could share and help each other. We didn’t share common responsibility, and therefore we lost the possibility for discussions and reflection. I believe we could have had more; we could have achieved better learning if we had worked differently.” Interview 14.*

## Discussion

The aim of the study was to describe, explore, and discuss how students undertaking an online course collaborated in small groups. Overall, students reported a positive experience of studying the philosophy of science and methods in an online context, and most of the students reported that working in small groups was essential for learning complex aspects of this subject. The participants in our study reported that group size (three–five) was a significant characteristic for the work process, along with a reciprocal blend of digital resources and assignments. These characteristics and continuity of group members throughout the online course made it possible to complete and deliver complex assignments. Our findings are in line with factors identified by Scager [20] as being important when working together. By working in small groups, the students experienced a level of support and understanding among their fellow students, and the fact that all assignments in the online course were group-based forced them to collaborate to achieve the learning outcomes.

Even though the students thought that CL was essential for their learning, not all reflected on the relationship between the working processes within the group and their learning. We found that the different working processes adopted during the online course could be differentiated into three main group working processes. These processes were not all focused on collaborative learning. Rather, some focused more on the students’ own learning and competencies. These findings are in line with those of Johnson et al. [13], who also found variations in small-group working. The three main working processes were consistent throughout the whole online course. Although some students felt that the work process could have been better, and that they therefore had to do additional work, they resisted changing the process in fear of ruining the atmosphere in the group.

Among the groups using working process number 1, two approaches to the organization of work could be observed, meaning that the students often had meetings, but how they met and collaborated was different. Some groups met and distributed the work, clarifying assignments, discussing different opinions and interpretations, and coming to an agreement and common understanding. They worked on their specific set of tasks and then met again to finalize the work and complete the assignment. The other approach was to have a discussion and work together towards completion of the assignment, changing the campus group to an online group, focusing on collaborating in real time. Students in groups using both forms worked together and discussed all parts of the tasks as a team to complete different aspects of the complex assignments As some students expressed; *“I experienced that I learned more by participating in discussions with my peers and solving tasks together than spending time alone with my books” Interview 13.* They involved themselves and their fellow students and were committed to working as a team and to the subject matter [20, 34].

In working process number 2 (individual responsibility, with flexible organization), students acted more as individual contributors than as team members. The students’ started their collaboration by dividing the assignment into different sub-assignments, with students taking individual responsibility for their task. Groups used a “stapler” (as described by Scager [20]), i.e., a group member who was responsible for integrating each student’s work into a group paper. The groups did not seek to establish common knowledge or a shared understanding of the topic, and each student had individual responsibility for seeking out the necessary knowledge to complete his or her contribution to the assignment. Due to a lack of continuity in their interaction and collaboration, these groups might have lost the potential learning effect of collaboration. Johnson and Johnson [35] have called this behaviour “pseudo learning”. Although a sense of team cohesiveness is maintained through equal contributions from each member and by agreeing on distribution of the workload, this method of organizing work does not ensure that students perceive their work as an activity which facilitates learning; rather, they see it more as a way to “get the job done”.

Students adopting working process number 3 (individual responsibility, with unorganized groups) were organized as a group but did not act as a group, and the groups did not organize themselves. Only one or a few students took responsibility and got involved in the work, while the other group members did not participate in small-group collaboration. The students who took responsibility worked and collaborated in many of the ways that the students in working process 1 did, taking responsibility, using available resources and completing the assignments. As stated in one of the interviews – “we were two who took responsibility, and we sent documents and discussions back and forth between the two of us. That worked very well, but the rest of the group were absent, and that felt wrong” Interview 11.

Bliss and Lawrence [36] claim that one of the biggest obstacles to group learning is students who do not participate. Our findings demonstrate that this can indeed be a problem. The students who became involved were deprived of the benefits that could be achieved through discussion. This has also been reported by Bliss and Lawrence [36] and Liu and Tsai [37]. On the other hand, these students became well acquainted with the subject matter and were able to complete the assignments.

Our findings suggest that how students perceive the subject they are studying is related to the importance of the subject. Another key factor affecting adoption of collaborative work practices is having a common understanding of the subject and students’ expectations regarding their own level of participation in the subject. The students who organized their work using working process number 1 had a common understanding of the collaboration within the group. This made the members aware of what to do and their expectations of one another. It is uncertain whether all members fully recognized the meaning of the objectives of the online course, but how the group organized the work could have led to a common understanding. There was a random composition of the groups, and how all members within one group perceived the objectives of the course is uncertain. It may have been the case that members in groups focusing on individual responsibility could initially have had the same opinions as those working with joint responsibility. It seems that how the group organized (or did not organize) the work affected the students’ understanding of the course in philosophy and science, and this understanding could have changed because of the different working processes. Lave and Wenger [26] stated that active participation in the social context creates what they do as a group. It is more likely that the working processes arose due to a lack of understanding of the consequences of different group working processes for both individual members’ and fellow students’ learning, and the strategies used by the group at the outset persisted throughout the whole online course. The students who used working process number 3 had also not clarified the working process in advance, and this way of organizing the group seemed to leave the responsibility to a few members and not the entire group.

All three working processes identified seem to be in line with the definition of collaborative learning that emphasizes collaboration (cooperation) to achieve common goals [12, 13]. It is uncertain whether all working processes facilitate an individual’s own learning and that of others in small groups equally well. When learning is understood as a dynamic social process where knowledge is considered a response to social interaction, and a prerequisite is discussion and reflection on everyone’s contributions, it seems that not all of the three working processes identified in our study can be understood as CL.

The working process joint responsibility and flexible organization encouraged discussion and reflection, with all group members developing an understanding of the assignments. Many studies have found that in-depth learning is achieved through discussion and reflection with peers [17–22], and we found that students working with joint responsibility acted in line with this definition of in-depth learning. In this working process, the students had shared responsibility for assignments and a common understanding of the work, with all participants acquiring greater knowledge and understanding of all parts of the tasks. Through sharing responsibility, group discussions and feedback, the students not only acted as a group but more like a team. Bang and Midelfart [38] define a team as a group that has a task for which the group members are collectively responsible and where they are interdependent.

The three different collaboration processes adopted during the online course that we studied do not differ significantly from how students collaborate in in-person courses as shown in the literature [13, 19–22]. It seems that students adopt the same patterns and structures independently from the context they operate in. This could indicate that students collaborate and are influenced by the same factors regardless of where the collaboration takes place.

### Strengths and limitations

This case study gave us the opportunity to explore, in depth and over time, students’ experience of collaborative learning in small groups in online courses. Since there is little research (to the best of our knowledge) on how students learn in online subjects that are part of an otherwise campus-based education, it was important for us to gain an insight into different approaches to collaboration. This study was based on semi-structured interviews with students in relation to one case. We have described the case thoroughly so that readers can understand and recognize the parameters and relate them to their own situation [20, 39]. In this way, the findings may be useful in designing and implementing similar online courses. The small number of students in some of the focus groups and the fact that the students came from the same programme could be a limitation of the study. Students from the same programme may have been too self-conscious to reveal certain aspects of group work in front of their peers. Some students mentioned this, so we conducted individual interviews. However, there may have been students who also felt the same without saying so. Having only two to five students in each group did not enable the full benefits of the group processes to be revealed in such groups [32]. Author IR was present during the focus groups interviews as a co-moderator. If the students knew that he was one of the course designers and the person who solved their technical problems this may have influenced the participants’ responses.

Another strength, yet also a possible weakness, of the study is that KAA and IR were the ones who developed the online course. This gave them an understanding of the challenges and strengths of the course, but at the same time, they had to work to maintain an analytical distance from the data. The third researcher was not directly involved in development of the course and could therefore view the data impartially.

## Conclusions

This study contributes to knowledge of how students working in groups approach learning and identifies important factors about collaborative learning during online courses. This knowledge may be useful for educators designing and facilitating online courses and for instructors supervising groups. This study shows that even if design factors are the same (e.g., group size, challenging and relevant assignments, and student autonomy in terms of being able to organize group working processes), the working process that each group chooses can differ.

Although the identified working processes were found to promote collaboration only one working process promoted group discussion of all parts of the tasks, working as a team completing different aspects of complex assignments. Future online teaching might require an even stronger focus on students’ internal motivation for learning and the importance of teacher presence and teachers’ ability to facilitate online education.

## Data Availability

The datasets generated and/or analysed during the current study are not publicly available due to the confidentiality of the participants but are available from the corresponding author on reasonable request and with permission of the participants.

## Abbreviations

CL: Collaborative learning
MaMet: Online course in the philosophy of science and methods
ECTS credits: European Credit Transfer and Accumulation System
